# The role of ubiquitination and deubiquitination in cancer metabolism

**DOI:** 10.1186/s12943-020-01262-x

**Published:** 2020-10-01

**Authors:** Tianshui Sun, Zhuonan Liu, Qing Yang

**Affiliations:** 1 Department of Obstetrics and Gynecology, Shengjing Hospital of China Medical University, No. 36, Sanhao Street, Heping District, Shenyang, 110004 China; 2 Department of Urology, First Hospital of China Medical University, Shenyang, China

**Keywords:** Ubiquitination, Deubiquitination, Cancer, Metabolic reprogramming

## Abstract

Metabolic reprogramming, including enhanced biosynthesis of macromolecules, altered energy metabolism, and maintenance of redox homeostasis, is considered a hallmark of cancer, sustaining cancer cell growth. Multiple signaling pathways, transcription factors and metabolic enzymes participate in the modulation of cancer metabolism and thus, metabolic reprogramming is a highly complex process. Recent studies have observed that ubiquitination and deubiquitination are involved in the regulation of metabolic reprogramming in cancer cells. As one of the most important type of post-translational modifications, ubiquitination is a multistep enzymatic process, involved in diverse cellular biological activities. Dysregulation of ubiquitination and deubiquitination contributes to various disease, including cancer. Here, we discuss the role of ubiquitination and deubiquitination in the regulation of cancer metabolism, which is aimed at highlighting the importance of this post-translational modification in metabolic reprogramming and supporting the development of new therapeutic approaches for cancer treatment.

## Background

Metabolic pathways are of vital importance in proliferating cells to meet their demands of various macromolecules and energy [1]. Compared with normal cells, cancer cells own malignant properties, such as increased proliferation rate, and reside in environments short of oxygen and nutrient. Correspondingly, metabolic activities are altered in cancer cells to support their malignant biological behaviors and to adapt to stressful conditions, such as nutrient limitation and hypoxia [1]. Cancer metabolism is an old field of research. Warburg effect observed in the 1920s provides a classical example of metabolic reprogramming in cancer [2]. In the past few decades, enhanced biosynthesis of macromolecules, altered energy metabolism, and maintenance of redox homeostasis have been observed to be essential features of cancer metabolism. Altered metabolism in cancer cells have aroused increasing attention and interest [3]. Because of the generality of metabolic alterations in cancer cells, metabolic reprogramming is thought as hallmark of cancer, providing basis for tumor diagnosis and treatment [1]. For instance, the application of 18F-deoxyglucose positron emission tomography is based on tumor cells’ characteristic of increased glucose consumption [4]. Inhibition of some metabolic enzymes, such as L-lactate dehydrogenase A chain (LDH-A), have been observed to regress established tumors [5, 6]. Therefore, research of metabolic reprogramming is of critical importance, which might provide new opportunities for cancer diagnosis and treatment.

Amongst multiple post-translational modification, protein ubiquitination is a common and important process in cells [7, 8]. Ubiquitination and deubiquitination have been observed to be dysregulated in various types of cancers. Genetic and epigenetic aberrations, such as mutation, amplification and deletion, can be the common causes of dysregulated ubiquitination and deubiquitination in cancer cells [9]. Ubiquitination and deubiquitination can also be abnormally regulated by transcriptional, translational or posttranslational mechanisms in cancer cells, exerting oncogenic or anti-cancer roles in carcinogenesis [7, 8, 10]. In recent years, the involvement of ubiquitination and deubiquitination in the regulation of metabolic reprogramming in cancer cells has received a growing body of attention [11]. Given the complexity and importance of both cancer metabolism and protein ubiquitination, the exact roles of protein ubiquitination and deubiquitination in metabolic reprogramming are worth further studies and analyses. The present review will highlight the ubiquitination and deubiquitination system as a regulator of cancer metabolism and discuss future directions focusing on the strategies to improve cancer therapy.

## Cancer metabolism

To satisfy nutrient and energy requirements for cells’ survival and growth, metabolic pathways are altered in cancer cells, which is called metabolic reprogramming [1]. Metabolic reprogramming is a highly regulated process [12]. Aberrant activation of mechanistic target of rapamycin complex 1 (mTORC1) is one of the most common alterations in proliferating cancer cells, playing a key role in metabolic reprogramming [12]. Under the stimulation of amino acids, mTORC1 can be activated, which subsequently exerts various biological effects by activation of different downstream targets, such as hypoxia-inducible factor 1 (HIF-1) and sterol regulatory element-binding protein (SREBP) [12, 13]. Proliferating cancer cells require elevated synthesis of protein, lipid and nucleotide. Glycolysis can be upregulated by mTORC1 activation, providing more glycolytic intermediates for biosynthesis of these macromolecules [14]. Moreover, mTORC1 activation promotes glutamine uptake to maintain mitochondrial ATP production [15]. Fatty acids can also supply carbon to the tricarboxylic acid (TCA) cycle to sustain mitochondrial function [16]. PI3K-AKT signaling is the most well-known mechanism for activating mTORC1 [17]. Besides, mTORC1 can be activated or inhibited by various signaling pathways directly or indirectly. For instance, 5′-AMP-activated protein kinase (AMPK) activated by energy shortage is a crucial inhibitor of mTORC1 [18]. What’s more, transcription factor c-Myc and p53 also take part in metabolic reprogramming through transcriptional regulation of metabolism-related genes [19, 20]. Based on multiple regulatory mechanisms, expression or activity of the enzymes involved in glucose, amino acids and fatty acids metabolism are altered, directly contributing to metabolic reprogramming [21]. What’s more, the up-regulation of various metabolic processes in cancer cells triggers accumulation of reactive oxygen species (ROS) [22]. Transcription factors nuclear factor erythroid 2-related factor 2 (NRF2) and HIF-1 play key roles in maintenance of redox homeostasis, keeping ROS in an appropriate level to promote tumor growth rather than inducing damage [23, 24].

Under nutrient rich conditions, activation of mTORC1 supports cancer cells growth. In periods of cellular stress, low levels of amino acids or absent ATP induces mTORC1 inhibition, which subsequently activates a compensatory mechanism named autophagy [25]. Autophagy is a highly regulated pathway essential for cell survival in nutrient-deprived conditions, complementing the classical pathways like glycolysis. Autophagy supplies amino acids by inducing degradation of macromolecules and organelles in lysosome, thereby providing intracellular amino acids supply to fuel the TCA cycle, gluconeogenesis and protein synthesis [26]. However, the interplay between autophagy and glycolysis seems to be complex. Activation of autophagy has been observed to enhance glycolysis [27]. Deficiency of mitophagy can induce mitochondrial dysfunctions, enhancing glycolysis and Warburg effect [28]. Additionally, studies have found that oxidative stress induced by cancer cells can promote aerobic glycolysis and autophagy in cancer associated fibroblasts to obtain recycled nutrients from cancer associated fibroblasts. This phenomenon is called “Reverse Warburg Effect” [29]. Therefore, both the anabolic pathways, such as glycolysis, and the catabolic pathways, such as autophagy, interplay with each other, together contribute to cancer metabolism and supporting cellular growth. Taken together, abnormal alterations of multiple signaling pathways, transcription factors and metabolic pathways synergistically lead to metabolic reprogramming in cancer cells.

## Ubiquitination and deubiquitination

Ubiquitination is an ATP-dependent cascade process ligating ubiquitin, a ubiquitously expressed protein consisting of 76 amino acids, to a substrate protein [30]. Ubiquitin-activating enzymes (E1s) initially bind to ubiquitin for activation, and then transfer activated ubiquitin to ubiquitin-conjugating enzymes (E2s). Ubiquitin ligases (E3s) finally transfer ubiquitin from E2 to substrates [30]. According to the number of ubiquitin attaching to one lysine residue in protein, ubiquitination is divided into monoubiquitination (single ubiquitin) and polyubiquitination (a chain of ubiquitin) [31]. In the polyubiquitination chain, ubiquitin can be attached via 7 lysine residues (K6, K11, K27, K29, K33, K48, and K63) or the first methionine (M1) [32]. Different types of ubiquitination lead to disparate fates of substrate proteins. K48-linked polyubiquitination is the most widely studied type, which mainly labels proteins for 26S proteasome-mediated recognition and degradation [32]. K48-linked polyubiquitination also has proteasome independent functions, including regulation of signaling events and transcription, which are possibly determined by the length of the ubiquitin chain [33–35]. K11-linked polyubiquitination is also associated with proteolysis [32]. Ubiquitin-proteasome system is involved in the degradation of more than 80% of proteins in cells [36]. K63-linked polyubiquitination is involved in signaling assemblies [32]. E3 ligases play a key role in the whole process of ubiquitination because of their specificity for substrates. In human genome, there are approximately 1000 E3 ligases, which can be divided into the homology to E6AP C terminus (HECT) domain-containing E3s, the RING-between-RING (RBR) family E3s and the really interesting new gene (RING) finger domain-containing E3s [37]. Deubiquitination is catalyzed by deubiquitinating enzymes (DUBs) to remove ubiquitin from ubiquitinated proteins, thus reversing the ubiquitination process [7]. About 100 DUBs fall into seven subgroups: the ubiquitin-specific proteases (USPs), the ubiquitin C-terminal hydrolases (UCHs), the ovarian tumor proteases (OTUs), the Machado-Josephin domain proteases (MJDs), the JAB1/MPN+/MOV34 (JAMM) domain proteases, the monocyte chemotactic protein-induced proteins (MCPIPs), and the motif interacting with ubiquitin-containing DUB family (MINDY) [10]. Dynamic conversion between ubiquitination and deubiquitination is closely related to various cellular functions and thus, its dysregulation results in multiple disease, such as neurodegenerative diseases and cancer [38]. Understanding of ubiquitination and deubiquitination may provide novel insights into the treatment of these diseases.

## Ubiquitination and metabolic signaling pathways

### Ubiquitination of mTOR

**Fig. 1:**
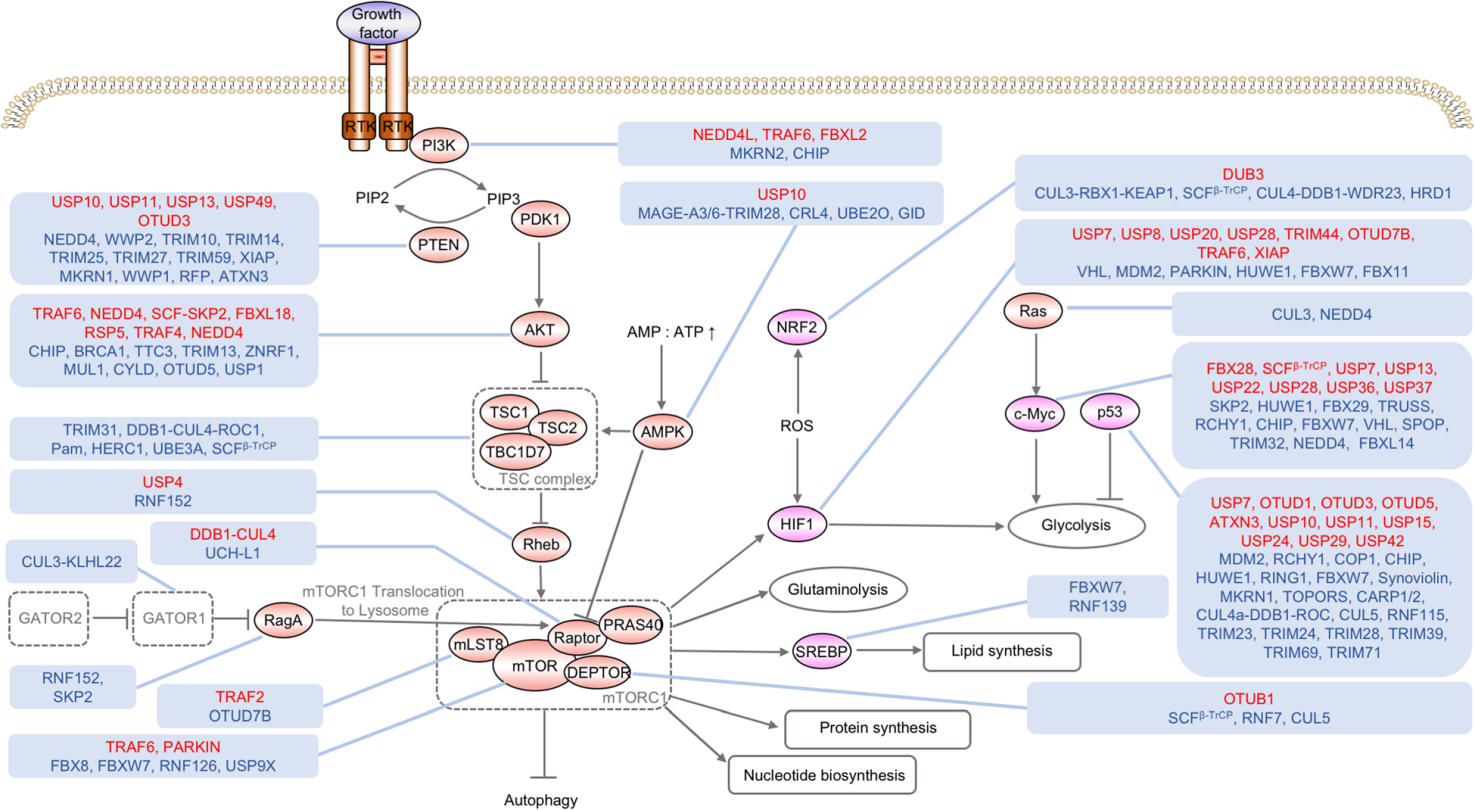
Regulation of signaling pathways and transcription factors associated with cancer metabolism by ubiquitination and deubiquitination. Aberrant activation of signaling pathways like PI3K-AKT-mTORC1, loss of tumor suppressive transcription factors like p53 and activation of oncogenic transcription factors like c-Myc control cancer metabolism. Ubiquitination and deubiquitination indirectly regulate cancer metabolism by modulating these signaling molecules and transcription factors. The ubiquitin ligases and deubiquitinating enzymes in red font positively regulate the activity or expression level of substrate proteins. The ubiquitin ligases and deubiquitinating enzymes in blue font negatively regulate the activity or expression level of substrate proteins. AMP, adenosine monophosphate; ATP, adenosine triphosphate; mTORC1, mTOR complex 1; PIP2, phosphatidylinositol 4,5-bisphosphate; PIP3, phosphatidylinositol (3,4,5)-trisphosphate; ROS, reactive oxygen species; RTK, receptor tyrosine kinase; TSC, tuberous sclerosis complex

Aberrant activation of mTORC1 is considered as a key feature of metabolic reprogramming. mTORC1 is a complex consisting of mTOR, Raptor, mLST8, PRAS40 and DEPTOR [39]. mTOR is an evolutionarily conserved serine/threonine protein kinase in the PI3K-related kinase superfamily, responsible for the catalytic activity of mTORC1 [40]. Translocation of mTORC1 to lysosome is the premise for its subsequent activation, identified as a critical step in the activation of mTORC1 signaling [41]. Activated RagA is thought to be the main participator in the re-localization of mTORC1 to the lysosomes in amino acid-stimulated cells [41]. Studies have found that E3 ligase TRAF6, which is upregulated in cancer cells, can mediate K63-linked polyubiquitination of mTOR by interacting with p62 under the stimulation of amino acids, promoting the translocation of mTORC1 to the lysosomes and subsequent activation (Fig. 1) [42]. In addition, decreased K48-linked ubiquitination of mTOR by E3 ligase FBX8 and FBXW7 alleviates proteasome-dependent degradation of mTOR, exerting an oncogenic effect in cancer as well [43, 44]. Reduced mTOR ubiquitination is also linked to therapy resistance in cancer. Everolimus is a mTOR inhibitor used in breast cancer patients. Following downregulated phosphorylation of mTOR induced by depletion of dual specificity tyrosine-phosphorylation-regulated kinase 2 (DYRK2), ubiquitination and degradation of mTOR diminish, resulting in everolimus resistance [45]. Non-thermal plasma exerts anti-tumor effect by inducing RNF126 mediated K48-linked polyubiquitination and degradation of mTOR [46]. However, when faced with mitochondrial stress, E3 ligase PARKIN targets mTOR for ubiquitination, which maintains mTORC1 activity instead of affecting mTOR stability, thereby enhancing cell survival [47]. DUB USP9X can negatively modulate mTOR function and mTORC1 activity without changing mTOR protein level [48]. Therefore, different types of ubiquitination and deubiquitination play diverse roles in the regulation of mTOR function.

### Ubiquitination of raptor

Raptor is the regulatory protein of mTORC1, maintaining the correct subcellular localization of mTORC1 and allowing the binding of mTORC1 with substrates [49]. DDB1-CUL4 E3 ligase complex is essential for maintaining mTORC1 stability by ubiquitinating Raptor. Displacement of DDB1-CUL4 complex by DUB UCH-L1 can remove K63-linked poly-ubiquitin chains on Raptor, bringing about reduced mTORC1 [49].

### Ubiquitination of mLST8

mLST8, also called GβL, is a component of both mTORC1 and mTORC2. mLST8 is associated with the catalytic domain of mTOR and stabilizes its kinase activation loop [50]. mLST8 can be ubiquitinated by TRAF2 through K63-mediated linkage, which breaks the interaction between mLST8 and SIN1 in mTORC2, giving rise to elevated formation of mTORC1. This process can be reversed by DUB OTUD7B, leading to increased mTORC2 formation [50]. The study highlighted the role of ubiquitination and deubiquitination in the balance and competence between mTORC1 and mTORC2 signaling under various conditions.

### Ubiquitination of DEPTOR

DEPTOR is an inhibitor of both mTORC1 and mTORC2. mTOR activation can phosphorylate DEPTOR and promote its recognition by SCF^β-TrCP^ ubiquitin ligase, targeting DEPTOR for polyubiquitination and proteolytic degradation [51, 52]. In tumors with isocitrate dehydrogenase1/2 (IDH1/2) mutations, oncometabolite 2-hydroxyglutarate indirectly promotes DEPTOR polyubiquitination by SCF^β-TrCP^, thus activating mTOR [51]. E3 ligase RNF7 exerts oncogenic effect in prostate tumorigenesis by promoting ubiquitination and degradation of DEPTOR [53]. DUB OTUB1 specifically stabilizes DEPTOR via deubiquitination, thereby playing an anticancer role [54]. CUL5 also targets DEPTOR for degradation, which is inhibited during autophagy activation [55]. Autophagy triggered by decreased ubiquitination and degradation of DEPTOR is related to drug resistance in cancer. Anticancer agent MLN4924 causes protective autophagy via inactivation of the CUL E3 ligase and accumulation of DEPTOR, which suppresses its effectiveness [56]. Enhancement of DEPTOR degradation can attenuate autophagy, which has been found to be an effective target in Temozolomide-resistant glioblastoma cells [57].

### Ubiquitination of RagA

As we mentioned above, activated RagA plays a key role in the re-localization of mTORC1 to the lysosomes and subsequent activation of mTORC1 [41]. Study found that downregulation of lysosomal E3 ligase RNF152 protected cells from autophagy [58]. RNF152 modifies RagA by K63-linked polyubiquitination and promotes recruitment of RagA inhibitor GATOR1, thus inducing RagA inactivation. Then mTORC1 is released from the lysosomal surface, giving rise to blockade of mTORC1 signaling pathway [58]. Moreover, K63-linked polyubiquitination of RagA can also be mediated by E3 ligase SKP2, which exerts a similar effect with RNF152 [59].

### Ubiquitination of GATORs

GATOR1 is a complex consisting of DEPDC5, NPRL2 and NPRL3, while GATOR2 consists of Mios, WDR24, WDR59, Seh1L and sec13. GATOR2 negatively regulates DEPDC5 in GATOR1, which acts as an inhibitor of RagA. Thus, GATOR2 exerts a promoting effect on RagA. Oncogenic E3 ligase CUL3-KLHL22 was observed to mediate K48-linked polyubiquitination of DEPDC5 and target DEPDC5 for degradation under the stimulation of amino acids and promote mTORC1 activation in tumor [60].

### Ubiquitination of Rheb

GTP-bound Rheb is the activator of mTORC1 after translocation of mTORC1 to the lysosome. Monoubiquitination of Rheb by the lysosomal E3 ligase RNF152 can enhance its interaction with the tuberous sclerosis complex (TSC) complex, the major inhibitor of Rheb, and can decrease mTORC1 activation [61]. Following phosphorylation by AKT, USP4 can deubiquitinate Rheb to reverse the action of RNF152. Therefore, the dynamic change of ubiquitinating state of Rheb is associated with mTORC1 activation and tumor growth [61].

### Ubiquitination of TSC complex

TSC complex, composed of TSC1, TSC2, and TBC1D7, is identified as a major upstream regulator of Rheb, converting GTP-bound Rheb to GDP-bound Rheb, thus inhibiting mTORC1 activation. TRIM31 mediated K48-linked ubiquitination of TSC1-TSC2 complex induces its degradation and promotes growth of hepatocellular carcinoma cells [62]. FBXW5 recruits TSC2 protein to DDB1-CUL4-ROC1 E3 ligase complex, promoting its ubiquitination and degradation [63]. Moreover, E3 ligase Pam, HERC1 and UBE3A were also observed to mediate TSC2 protein ubiquitination and to enhance its degradation [64–66]. Interaction of TSC2 with TSC1 can prevent TSC2 from HERC1 ubiquitin ligase mediated degradation [66], while VPS34 can competitively bind to TSC1, resulting in TSC2 degradation [67]. E3 ubiquitin ligase SCF^β-TrCP^ can ubiquitinate free TBC1D7 for degradation. Binding to TSC or AKT dependent phosphorylation of free TBC1D7 can prevent TBC1D7 from interaction with SCF^β-TrCP^, stabilizing the pool of TBC1D7 [68].

### Ubiquitination of PI3K

PI3K-mTORC1 signaling is the crucial signaling pathway that governs metabolic reprogramming and tumor cell growth. PI3K can be activated under the stimulation of growth factors. Activated PI3K phosphorylates PIP2, converting PIP2 to PIP3, which recruits 3-phosphoinositide-dependent protein kinase 1 (PDK1) and Akt to the membrane. PDK1 subsequently activates AKT, a negative regulator of TSC complex, and subsequently activating mTORC1 [69]. Activation of PI3K-mTORC1 signaling can exert various effects on metabolic process and play a key role in the regulation of tumor metabolism [69]. PI3K is a dimeric enzyme composed of a catalytic subunit (p110α, p110β, or p110γ) and a regulatory subunit (p85α, p85β, p55α, p55γ, or p50α) [70]. A dynamic cycle of proteasome-dependent degradation and re-synthesis of PI3Kp110α were observed in activation of PI3K signaling. NEDD4L E3 ligase catalyzes free PI3Kp110α for ubiquitination, leading to its proteasome-dependent degradation and maintenance of PI3K signaling [70]. TRAF6 E3 ligase promotes activation of PI3K pathway in cancer by nonproteolytic polyubiquitination of PI3K catalytic subunit p110α [70]. TRAF6 also directs PI3K recruitment to TGF-β receptor via K63-linked polyubiquitination of PI3Kp85α, which is essential for TGF-β-induced activation of PI3K signaling [71]. What’s more, PI3K regulatory subunit p85α can be ubiquitinated by E3 ligase MKRN2 and E3 ligase complex HSP70-CHIP through K48-mediated linkage, bringing about proteolysis of PI3Kp85α and downregulation of PI3K signaling in cancer [72, 73]. Dephosphorylated free p85β is ubiquitinated by FBXL2 for proteolysis. Decreased free p85β reduces its competition with p85-p110 heterodimers for docking sites on cell membrane, thus upregulating PI3K signaling [74]. Therefore, both the catalytic subunit and the regulatory subunit can be ubiquitinated, exerting various effects on PI3K signaling.

### Ubiquitination of PDK1

PDK1 phosphorylates and activates AKT, transducing signal from activated PI3K to AKT. Attenuated ubiquitination and degradation of PDK1 are related to chemoresistance in ovarian cancer [75]. Monoubiquitination of PDK1 in cancer cell lines can be reversed by USP4 catalyzation, the function of which still remains unclear [76].

### Ubiquitination of AKT

AKT, a negative regulator of TSC complex, can be activated by PI3K signaling, exerting oncogenic effect by promoting mTORC1 signaling. K63-linked polyubiquitination of AKT catalyzed by TRAF6, NEDD4, SCF-SKP2, FBXL18, RSP5 or TRAF4 E3 ligase is required for cell membrane localization of AKT and is essential in activation of PI3K-AKT-mTOR pathway and subsequent upregulation of glycolysis [77–82]. SETDB1 catalyzed methylation of AKT enhances its K63-linked ubiquitination and activation [83]. After interaction with KDM4B, TRAF6 promotes its ubiquitination of AKT in colorectal cancer, facilitating glucose metabolism and tumor growth [84]. DUB CYLD, OTUD5 and USP1 can reverse K63-linked polyubiquitination of AKT and inhibit its activation [85–87]. Bisdemethoxycurcumin can inhibit hepatocellular carcinoma cell growth by promoting CYLD-mediated deubiquitination of AKT [88]. Enhanced AKT ubiquitination and activation caused by downregulation of DUB OTUD5 give rise to radioresistance in cervical cancer [86]. pAKT ubiquitinated by NEDD4 can regulate its nuclear trafficking to promote tumorigenesis [79]. What’s more, E3 ligases CHIP, BRCA1, TTC3, TRIM13, ZNRF1, MUL1 can modify AKT by K48-linked ubiquitination and promote degradation of AKT to suppress its activation [89–94]. Anticancer agents *Rhus coriaria*, SC66 and Vitamin C can stimulate ubiquitination and degradation of AKT in cancer cells [95, 96]. When proteasome impairs in cellular stress, E3 ligase MUL1 catalyzes K48-linked ubiquitination of AKT, which subsequently undergoes lysosomal degradation, playing key roles in cellular survival [97]. This process can be reversed by DUB USP7 [97].

### Ubiquitination of PTEN

As a negative regulator of PI3K/AKT signaling pathway, phosphatase and tensin homolog (PTEN) converts PIP3 back to PIP2, playing tumor suppressive functions in various cancers. Identified as the E3-ligase of PTEN, NEDD4 not only contributes to monoubiquitination of PTEN for its nuclear transport but also mediates polyubiquitination of PTEN for its proteasomal degradation to activate AKT signaling transduction [98]. Up-regulation of LINC00152 enhances NEDD4 mediated ubiquitination and degradation of PTEN in breast cancer [99]. E3 ligase WWP2, TRIM10, TRIM14, TRIM25, TRIM27, TRIM59 and XIAP can target PTEN for ubiquitination and degradation as well [100–106]. AKT activated MKRN1 E3 ligase also mediates ubiquitination and degradation of PTEN, thus positively regulating PI3K/AKT signaling axis [107]. The E3 ligase WWP1 mediated polyubiquitination can suppress the membrane recruitment and function of PTEN [108]. Ubiquitination via E3 ligase RFP can downregulate PTEN phosphatase activity rather than altering its stability or localization [109]. As for the deubiquitination of PTEN, USP10, USP11, USP13, USP49, and OTUD3 catalyze removal of K48-linked ubiquitin chain on PTEN to enhance protein stability of PTEN and attenuate AKT signaling pathway [110–114]. USP7-induced deubiquitination of PTEN results in its nuclear exclusion rather than regulating its protein stability [115, 116]. DUB ATXN3 suppress PTEN expression by reducing its transcription rather than altering its protein level [117].

### Ubiquitination of AMPK

AMPK is an inhibitor of mTORC1 by phosphorylating Raptor and activating TSC2. As an energy sensor, AMPK is crucial in maintenance of NADPH and ATP level in response to reduced intracellular ATP. AMPK is composed of catalytic α and regulatory β and γ subunits [118]. E3 ligase complex MAGE-A3/6-TRIM28 and E3 ligase CRL4A catalyze ubiquitination of AMPKα and target it for degradation, thus reducing autophagy and altering cancer metabolism [118, 119]. UBE2O, an atypical ubiquitin enzyme with both E2 and E3 activities, ubiquitinates AMPKα2 for degradation [120]. CRL4 catalyzed ubiquitination also directs AMPKγ proteolysis [121]. GID ubiquitin ligase mediates ubiquitination and degradation of AMPK as well, leading to decreased autophagy and increased mTOR activity [122]. Ubiquitination of AMPKα can be reversed by USP10 to remove the ubiquitin chain from AMPKα and promote AMPK activation [122].

### Ubiquitination of KRAS

As a common mutated oncogene driving tumorigenicity in pancreatic, colon and lung cancers, KRAS enhances expression of glucose transporter type 1 (GLUT1) and controls glycolysis and glutamine metabolism in cancer cells, which is considered to be associated with metabolic reprogramming in primary invasive cancers [123]. Monoubiquitination and diubiquitination of KRAS elevate its GTP loading ability [124]. CUL3-based E3 ligase complex can mediate polyubiquitination and degradation of KRAS [125]. KRAS4B, an alternative splicing of KRAS gene, is targeted by E3 ligase NEDD4 for ubiquitination and proteolysis. However, activated KRAS signaling upregulates NEDD4 expression and prevents NEDD4-mediated KRAS ubiquitination. This in return promotes NEDD4 catalyzed degradation of PTEN to trigger tumor growth [126]. Therefore, modification of signaling molecules by ubiquitination can exert various effects in signaling pathways, thereby regulating metabolic reprogramming in cancer cells.

## Ubiquitination and transcription factors

### Ubiquitination of HIF-1

HIF-1 is a metabolic-associated transcription factor which can be activated by mTORC1, accumulation of ROS and accumulation of TCA cycle metabolites. Activation of HIF-1 enhances expression of various glycolytic genes including hexokinase 1 (HK1), HK2, LDHA, and pyruvate dehydrogenase kinase isoform1 (PDK1), strengthening glycolytic flux and maintaining redox homeostasis [127]. HIF-1 consists of α and β subunits. Compared with HIF-1β, HIF-1α is unstable and susceptible to ubiquitination. E3 ligase VHL can mediate degradation of HIF-1α under normoxic conditions. Loss of VHL in cancer cells stabilizes HIF-1α, contributing to aerobic glycolysis [128, 129]. E3 ligase MDM2, PARKIN and HUWE1 can catalyze ubiquitination of HIF-1α and targets it for degradation, thus exerting an anti-tumor effect [130–132]. Tumor suppressors PTEN and p53 can enhance MDM2-mediated ubiquitination and degradation of HIF-1α to inhibit tumorigenesis [133–135]. Following phosphorylation by GSK3β, HIF-1α ubiquitination via FBXW7 is increased, which promotes proteolysis of HIF-1α and inhibits tumor growth [136]. Anticancer drug glyceollins and thymoquinone can inhibit tumor growth by elevating ubiquitination and degradation of HIF-1α [137, 138]. E3 ligase FBX11 can reduce the mRNA stability rather than protein stability of HIF-1α [139]. DUB USP28 can antagonize the ubiquitination of HIF-1α by FBXW7 [136]. TRIM44, USP20 and USP7 also stabilizes HIF-1α by deubiquitination, leading to tumor progression under hypoxia [140–143]. DUB OTUD7B can suppress degradation of HIF-1α via proteasome-independent manner [144]. USP8 mediated deubiquitination of HIF-1α was to maintain its expression level in normal conditions [145]. K63-linked polyubiquitination of HIF-1α catalyzed by TRAF6 can protect it from degradation [146]. XIAP modifies HIF-1α by K63-linked polyubiquitination to promote its nuclear retention and enhance HIF-dependent gene expression [147]. In conclusion, ubiquitination is an important regulatory mechanism of HIF-1.

### Ubiquitination of c-Myc

Transcription factor c-Myc contributes to metabolic reprogramming through transcriptional regulation of genes participating in metabolism, such as LDHA [148]. c-Myc is an unstable protein susceptible to ubiquitination and proteolysis. E3 ligase SKP2, HUWE1, FBX29, TRUSS, RCHY1, CHIP, FBXW7, VHL, SPOP, TRIM32, NEDD4 and FBXL14 can target c-Myc for ubiquitination and degradation [149–160]. Decreased ubiquitination of c-Myc by VHL is observed to drive aerobic glycolysis in breast cancer cells [159]. Mutation or deletion of these E3 ligase genes induces carcinogenesis by attenuating c-Myc degradation. Anticancer drugs, including lanatoside C, diminish cancer cell growth by upregulating ubiquitination and degradation of c-Myc in cancer [161–163]. Ubiquitination of c-Myc can also be regulated by interaction with other molecules. FBXL16 can competitively bind with c-Myc without inducing ubiquitination of c-Myc, thereby rescuing c-Myc from FBXW7-mediated degradation [164]. Interaction with Evi5 also antagonizes FBXW7-mediated ubiquitination of c-Myc protein in laryngeal squamous cell carcinoma [165]. Phosphorylated ANXA2 interacts with MYC and inhibits ubiquitin-dependent proteasomal degradation of MYC protein in esophageal cancer [166]. LncRNA XLOC_006390, GLCC1 and LINC01638 also block ubiquitination of c-Myc [156, 167, 168]. What’s more, E3 ligase FBX28 mediated non-proteolytic ubiquitination of c-Myc can enhance c-Myc dependent transcription [169]. Ubiquitination of c-Myc by E3 ligase SCF^β-TrCP^ in G2 phase can stabilize c-Myc to facilitate recovery from an S-phase arrest [170]. Moreover, the DUB USP7, USP13, USP22, USP28, USP36 and USP37 stabilize c-Myc, thereby stimulating tumor growth [155, 157, 171–174].

### Ubiquitination of p53

As a critical tumor suppressor participating in cell cycle regulation and apoptosis, p53 was also clarified by recent studies of its participation in metabolic reprogramming. Loss of p53 induces enhancement of glycolysis and maintenance of redox homeostasis in cancer cells [175]. Monoubiquitination, K48-linked and K63-linked polyubiquitination were observed to be common post-translational modification of p53 protein. MDM2/MDMX complex is the major E3 ligases and the main negative regulator of p53, degrading p53 and decreasing transcription of p53 target genes [176]. At the same time, MDM2 is targeted by p53 to form a negative feedback loop for the dynamic regulation of p53 under stressed and unstressed conditions [177]. RCHY1, COP1, CHIP, HUWE1, RING1, FBXW7, Synoviolin, MKRN1, TOPORS, CARP1/2, CUL4a-DDB1-ROC, CUL5, RNF115, TRIM23, TRIM24, TRIM28, TRIM39, TRIM69, TRIM71 can also target p53 for K48-linked polyubiquitination and degradation [178–185]. K48-linked polyubiquitination of p53 can be regulated by various molecules. For instance, MAVS and ATF3 can stabilize p53 by preventing p53 from MDM2-mediated ubiquitination [186, 187]. ACP5 mediated p53 phosphorylation enhanced the ubiquitination and degradation of p53 [188]. p53 protein can be deubiquitinated by DUB OTUD1, OTUD3, OTUD5, ATXN3, USP10, USP11, USP15, USP24, USP29 and USP42 to enhance its function as tumor suppressor under the conditions of high carcinogenicity and genotoxicity [117, 189–198]. DUB USP7 can catalyze deubiquitination of p53 and upregulate level of p53 protein as well [199]. On the other hand, USP7 mediates deubiquitination and stabilization of MDM2 and MDMX, the major E3 ligase of p53 protein, thereby reducing the protein level of p53 [200, 201]. Studies have found that the binding of USP7 with MDM2 is much stronger than that with p53 [200]. USP7 is highly expressed in most cancers, such as breast cancer and colorectal cancer, and plays carcinogenic role by deubiquitinating MDM2 and MDMX [202, 203]. Application of USP7 inhibitors can activate the p53 signaling in cancer cells and play anti-cancer functions [204]. However, USP7 is downregulated in some tumors, such as pulmonary adenocarcinoma, and plays a tumor suppressive role in p53-dependent mechanism [205]. Therefore, USP7 acts in a content-dependent manner and has a paradoxical action on p53 according to different tissues. What’s more, CUL7-mediated K63-linked polyubiquitination of p53 and MDM2 or MSL2-mediated monoubiquitination of p53 are associated with its translocation to the cytoplasm [206]. Anticancer drug HLI98 inhibits p53 degradation via MDM2 to enhance its tumor suppressive function [207]. Therefore, it’s likely for p53 ubiquitination regulation to act as an effective therapeutic method for cancers.

### Ubiquitination of NRF2

Accumulation of ROS can also activate NRF2, another essential transcriptional factor for the maintenance of redox homeostasis. KEAP1, a substrate-specific adapter of a CUL3-RBX1 E3 ligase complex, is the binding partner of NRF2 and it negatively controls NRF2 stability. Under normal conditions, the CUL3-RBX1 mediates ubiquitination and degradation of NRF2. Electrophile metabolites formed in oxidative stress prevents CUL3-RBX1-KEAP1 complex from ubiquitinating NRF2, thereby stabilizing NRF2. NRF2 subsequently activates transcription of antioxidant proteins, such as GPXs and TXNs as well as enzymes involved in glutathione and NADPH synthesis [208]. p62, RMP and CDK20 can competitively bind with KEAP1 [209–211]. TRIM25 targets KEAP1 for ubiquitination and degradation [212]. DUB USP15 can stabilize KEAP1 [212]. These events will change the level of NRF2 and affect the transcriptional activity of NRF2 targeted genes. In non-small cell lung cancer, phosphorylation by BMP8A or interaction with PAQR4 can prevent ubiquitination of NRF2 by KEAP1 and stabilize NRF2, resulting in chemotherapy resistance [213, 214]. Besides CUL3-RBX1-KEAP1 complex, E3 ligase SCF^β-TrCP^, CUL4-DDB1-WDR23, HRD1 can also ubiquitinate NRF2 and regulate its stability [215–217]. DUB DUB3 removes ubiquitin chain on NRF2 and stabilize NRF2, leading to chemoresistance in colorectal cancer [218].

### Ubiquitination of SREBP1

SREBP1 is a transcription factor associated with lipogenic genes [219]. mTORC1 signaling induces enhanced activity of SREBP1 to meet the requirements for fatty acid in proliferating cancer cells [220]. Activation of SREBP1 upregulates fatty acid synthesis as well as lipid import from extracellular space [220]. After phosphorylated by GSK-3, SREBP1 is prone to ubiquitination by E3 ligase FBXW7 and degradation via the ubiquitin-proteasome system [221, 222]. Deacetylation of SREBP by SIRT1 also enhances its ubiquitination and proteolysis [223]. RNF139 can ubiquitinate precursor forms of SREBP1, thereby preventing SREBP1 synthesis [224].

## Ubiquitination and autophagy

### Ubiquitination of ULK1

In nutrient-deprived conditions, inhibition of mTORC1 subsequently activates autophagy, a highly regulated pathway essential for cell survival [25]. Autophagy complements amino acids by inducing degradation of macromolecules and organelles in lysosomes [26]. ULK1 complex, composed of the ULK1, ATG13 and FIP200, is directly regulated by mTORC1 and is required for autophagy induction. TRAF6 catalyzed K63-linked polyubiquitination of ULK1 can stabilize ULK1 to promote activation of autophagy, which is related to drug resistance in chronic myeloid leukemia patients [225]. Activating molecule in BECN1-regulated autophagy protein 1 (AMBRA1) is a cofactor that interacts with Beclin-1 to regulate autophagy. Decreased phosphorylation of AMBRA1 caused by mTORC1 inactivation will promote its interaction with TRAF6 to upregulate polyubiquitination of ULK1, thereby potentiating autophagy initiation [226]. TRIM16 and TRIM32 also target ULK1 for K63-linked polyubiquitination, which stabilizes ULK1 and increases its phosphorylating activity, respectively [227, 228]. DUB USP1 hydrolyzes the K63-linked ubiquitin chain on ULK1 [229]. Downregulation of USP24 also enhances ULK1 ubiquitination, thereby increasing protein stability and kinase activity of ULK1 [230]. These studies indicate the shift between ubiquitinated ULK1 and deubiquitinated ULK1 is essential for autophagy initiation.

Ubiquitination was also observed to be essential in the regulation of autophagy threshold during autophagy progression. NEDD4L-mediated ULK1 ubiquitination via K27 and K29-linkage assembly induces its proteolysis [231]. Decreased level of ULK1 activates transcription of ULK1 to maintain its basal protein level. Newly synthesized ULK1 will be deactivated by mTOR to ensure a safe threshold of autophagy [231]. CUL3-KLHL20 complex can also downregulate autophagy by targeting activated ULK1 for ubiquitination and proteolysis [232]. ULK1 can be deubiquitinated by USP20, which prevents it from degradation, maintaining its basal level required for the initiation of autophagy [233]. In prolonged starvation, the interaction between USP20 and ULK1 reduces to terminate autophagy [233]. In conclusion, ubiquitination and deubiquitination play essential functions in both autophagy initiation and autophagy progression. Modulating ubiquitination might be an effective treatment for chemoresistant patients with enhanced autophagy in cancer cells.

### Ubiquitination of class III PI3K complex

Class III PI3K complex is composed of Beclin-1, ATG14, VPS34 and AMBRA1, essential for the nucleation of the phagophore. Various factors interact with Beclin-1 to regulate autophagy signaling. BCL2 can suppress autophagy by inhibiting Beclin-1 [234]. Starvation induced K63-linked ubiquitination of Beclin-1 by TRAF6 or AMBRA1 can block its interaction with BCL2, thus activating autophagy [235, 236]. AMBRA1 targeted polyubiquitination of Beclin-1 can enhance its association with VPS34 to activate of VPS34 [236]. TRIM50 mediated K63-linked polyubiquitination of Beclin-1 promotes its activation by ULK1 and induces autophagy in starvation [237]. DUB A20 and USP14 limit autophagy by reversing K63-linked ubiquitination of Beclin-1 [235, 238]. NEDD4 and RNF216 ubiquitinate Beclin-1 through K11- and K48- mediated linkage, respectively, which promote its degradation [239, 240]. DUB including USP19, ATXN3, USP10 and USP13 can reverse K11- or K48-ubiquitination of Beclin-1 to rescue it from degradation [241–243]. Beclin-1 also increases deubiquitinating activities of USP10 and USP13, further enhancing autophagy by positive feedback [241].

FBXL20 mediates ubiquitination and proteasome degradation of VPS34 to inhibit autophagy [244]. ATG14 was observed to be ubiquitinated by ZBTB16-CUL3-ROC1 E3 ubiquitin ligase complex for degradation [245].

AMBRA1 is degraded via the action of E3 ligase CUL4 under normal conditions. Activation of ULK1 disassociates CUL4 from AMBRA1, causing stabilization of AMBRA1 to promote autophagy. Disassociation of AMBRA1 with CUL4 can promote AMBRA1 binding to CUL5 to inhibit CUL5-mediated DEPTOR degradation, thereby inducing autophagy [55]. CUL4 can re-associate with AMBRA1 to promote its proteolysis when autophagy terminates, thus regulating autophagy response [55]. What’s more, E3 ligase RNF2 can also catalyze K48-linked ubiquitination and proteolysis of AMBRA1, thus downregulating autophagy [246]. Therefore, all the components of the Class III PI3K complex can be regulated by ubiquitination, which exerts important effects on autophagy.

### Ubiquitination of WIPI2

WIPI2 is involved in an early step of the formation of preautophagosomal structures. mTORC1 can mediate phosphorylation of WIPI2. E3 ligase HUWE1 interacts with phosphorylated WIPI2 and catalyzes ubiquitination of phosphorylated WIPI2, which is subsequently targeted for proteolysis, thus inhibiting autophagy flux [247].

### Ubiquitination of ATG4

ATG4 contributes to LC3 processing, playing an essential role in the phagophore expansion and autophagosome completion. E3 ligase RNF5 targets ubiquitination and degradation of ATG4B, thus limiting autophagy flux. When cell starves, RNF5 de-associates with ATG4B to induce autophagy [248].

## Ubiquitination and metabolic enzymes

### Ubiquitination and glucose metabolism

**Fig. 2:**
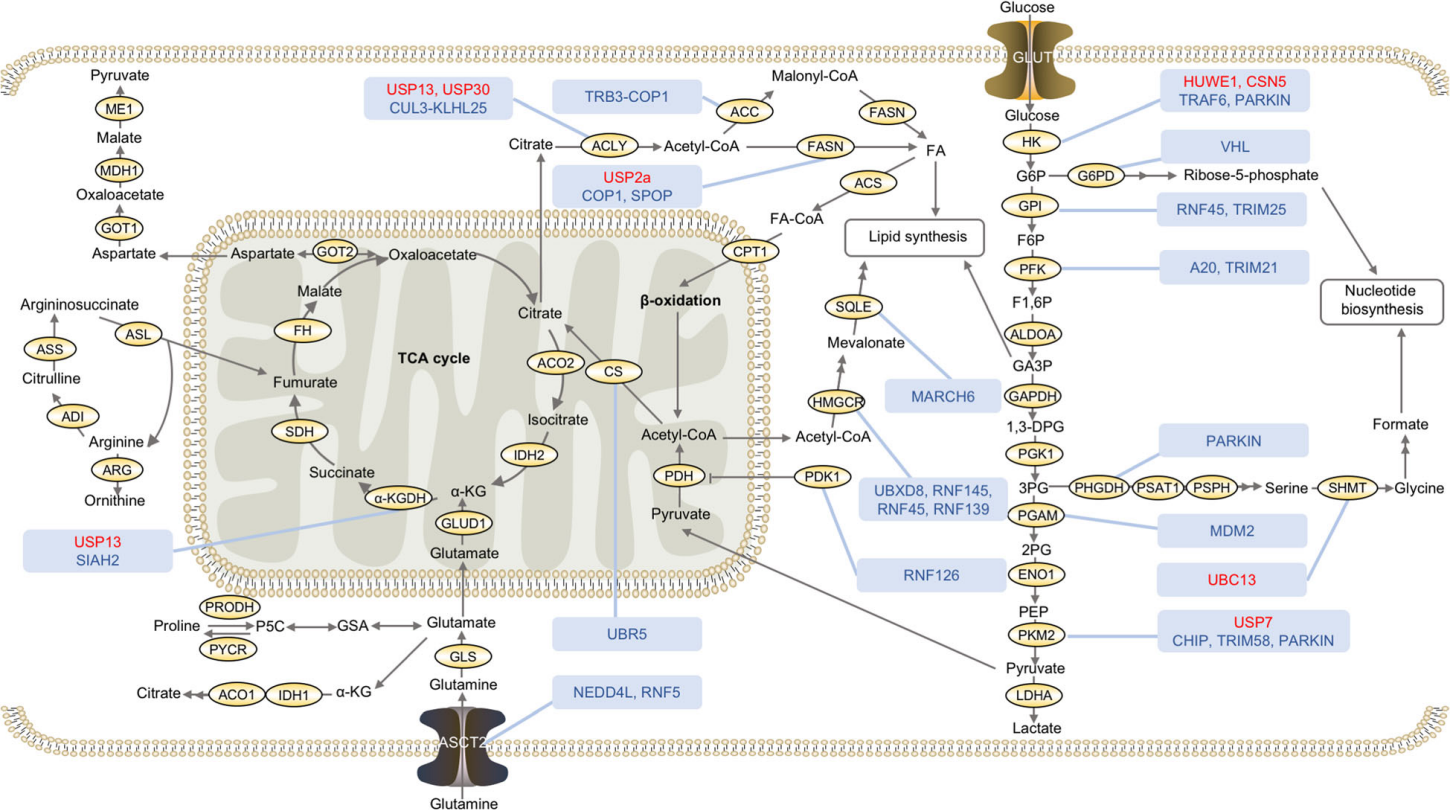
Regulation of metabolic enzymes by ubiquitination and deubiquitination in cancer metabolism. Glycolysis is upregulated to provide more glycolytic intermediates for biosynthesis of macromolecules. Glutamine uptake is enhanced to maintain mitochondrial ATP production. Fatty acids synthesis is increased for membrane biosynthesis. Metabolic enzymes involved in the glucose, fatty acid and amino acid metabolic pathways are under the regulation of ubiquitination and deubiquitination to control cancer metabolism. The ubiquitin ligases and deubiquitinating enzymes in red font positively regulate the activity or expression level of substrate proteins. The ubiquitin ligases and deubiquitinating enzymes in blue font negatively regulate the activity or expression level of substrate proteins. ACC, Acetyl-coenzyme A carboxylase; ACLY, ATP citrate lyase; ACO1/2, Aconitate hydratase 1/2; ACS, Acyl-CoA synthetase; ADI, Arginine deiminase; ALDOA, Fructose-bisphosphate aldolase A; ARG, Arginase; ASCT2, Neutral amino acid transporter B; ASL, Argininosuccinate lyase; ASS, Argininosuccinate synthase; CPT1, Carnitine O-palmitoyltransferase 1; CS, Citrate synthase; ENO 1, Enolase 1; FASN, Fatty acid synthase; FH, Fumarate hydratase; G6PD, Glucose-6-phosphate dehydrogenase; GAPDH, Glyceraldehyde-3-phosphate dehydrogenase; GLS1, Glutaminase 1; GLUD1, Glutamate dehydrogenase 1; GLUT1, Glucose transporter type 1; GOT, Aspartate aminotransferase; HK1, Hexokinase 1; HMGCR, 3-hydroxy-3-methylglutaryl-coenzyme A reductase; IDH1/2, Isocitrate dehydrogenase1/2; LDHA, L-lactate dehydrogenase A chain; MDH, Malate dehydrogenase; ME, NADP-dependent malic enzyme; PDH, Pyruvate dehydrogenase; PDK, Pyruvate dehydrogenase kinase; PFK, Phosphofructokinase; PFKFB3, 6-phosphofructo-2-kinase/fructose-2,6-bisphosphatase 3; PFKL, PFK liver type; PFKP, PFK1 platelet isoform; PGAM5, Phosphoglycerate mutase 5; PGK1, Phosphoglycerate kinase 1; PHGDH, D-3-phosphoglycerate dehydrogenase; PKM2, Pyruvate kinase M2; PRODH, Proline dehydrogenase; PSAT1, Phosphoserine aminotransferase 1; PSPH, Phosphoserine phosphatase; PYCR, Pyrroline-5-carboxylate reductase; SDH, Succinate dehydrogenase; SHMT1, Serine hydroxymethyltransferase 1; SQLE, Squalene epoxidase; TCA, Tricarboxylic acid; α-KGDH, α-ketoglutarate dehydrogenase

Increased glucose uptake and enhanced glycolytic flux are metabolic characteristics of cancer cells, supplying subsidiary pathways to provide precursors for macromolecule synthesis. Activated AKT was observed to inhibit ubiquitination of HK1, the first rate-limiting enzyme in the glucose metabolism pathway, promoting glycolysis and glioblastoma progression [249]. HUWE1 mediated K63-linked ubiquitination of HK2 promotes its re-localization and activation, enhancing glycolysis and tumor growth (Fig. 2) [250]. TRAF6 mediated K63-linked ubiquitination of HK2 directs HK2 degradation by autophagy, thereby negatively regulating glycolysis [251]. Deubiquitination of HK2 catalyzed by CSN5 can rescue it from degradation and enhance glycolytic flux during hepatocellular cancer metastasis [252]. Mitochondrial HK can also be ubiquitinated by PARKIN, inducing its proteasomal degradation [253].

Phosphofructokinase (PFK), the second rate-limiting enzyme in glycolysis, is a key regulator of glycolytic flux in cancer cells. Decreased A20 mediated ubiquitination and degradation of PFK liver type (PFKL) are related to increased glycolysis during hepatocellular carcinoma progression [254]. Phosphorylation of PFK1 platelet isoform (PFKP) by AKT can prevent it from TRIM21-mediated ubiquitination and degradation, promoting aerobic glycolysis in glioblastoma cells [255].

Pyruvate kinase M2 (PKM2) is the third rate-limiting enzyme of glycolysis. Decreased CHIP catalyzed ubiquitination and degradation of PKM2 are associated with Warburg effect in ovarian cancer cells [256]. Downregulated ubiquitination of PKM2 by TRIM58 is related to progression of osteosarcoma [257]. E3 ligase PARKIN modifies PKM2 by ubiquitination to decrease its enzymatic activity without affecting its stability [258]. PKM2 deubiquitinated by USP7 can strengthen the protein stability of PKM2 [259]. USP20 can also hydrolyze the ubiquitin chain on PKM2, but the detailed function of this deubiquitination is unclear [260].

Other enzymes in glucose metabolism can be also modified by ubiquitination. Glucose-6-phosphate isomerase (GPI), which catalyzes the conversion of glucose-6-phosphate to fructose-6-phosphate, can be ubiquitinated and degraded by E3 ligase RNF45 and TRIM25 [261]. 6-phosphofructo-2-kinase/fructose-2,6-bisphosphatase 3 (PFKFB3) is a glycolysis-promoting enzyme catalyzing the conversion between fructose 2,6-bisphosphate and fructose 6-phosphate. ROCK2 interacts with PFKFB3 and attenuates its ubiquitination and degradation in osteosarcoma cells [262]. What’s more, glycolysis during different stages of cell cycle in Hela cells is regulated by E3 ligases. Decreased E3 ligase APC/C-CDH1 can attenuate proteasomal degradation of PFKFB3, promoting glycolysis and transition of late G1 phase into S phase. E3 ligase SCF^β-TrCP^ activated during S phase can target PFKFB3 for degradation [263]. Similarly, decreased ubiquitination and increased stability of glutaminase 1 (GLS1) were also observed to be mediated by decreased APC/C-CDH1 in mid-to-late G1 [264]. Phosphoglycerate kinase 1 (PGK1) catalyzes the conversion of 1,3-diphosphoglycerate to 3-phosphoglycerate. Decreased ubiquitination of PGK1 leads to chemotherapy resistance in gallbladder cancer cells [265]. Interaction between MetaLnc9 and PGK1 blocks ubiquitin-mediated degradation of PGK1, promoting lung cancer metastasis [266]. Following phosphorylation, phosphoglycerate mutase (PGAM), which converts 3-phosphoglycerate to 2-phosphoglycerate, can be ubiquitinated by MDM2 for degradation [267]. Decreased ubiquitination of PGAM by MDM2 contributes to neoplastic transformation [267]. PDK can be ubiquitinated by RNF126, which targets them for proteasomal degradation, enhancing conversion of pyruvate to acetyl-CoA by pyruvate dehydrogenase (PDH) in cancer cells [268].

Ubiquitination of enzymes involved in the TCA cycle is also associated with cancer progression. Decreased UBR5-mediated ubiquitination of citrate synthase leads to citrate accumulation in hypoxia breast cancer cells, promoting cell migration, invasion, and metastasis [269]. HIF-1 activation under hypoxia condition can promote α-ketoglutarate dehydrogenase (α-KGDH) complex ubiquitination and proteolysis by SIAH2. Decreased α-KGDH activity inhibits glutamine oxidation and promotes glutamine-dependent lipid synthesis for tumor growth [270]. USP13 promotes ovarian cancer progression by deubiquitinating and upregulating α-KGDH [271].

Glucose-6-phosphate dehydrogenase (G6PD) catalyzes the oxidative pentose phosphate pathway, an essential process producing ribose-5-phosphate and NAPDH from G6P. G6PD was observed to be ubiquitinated and degraded by VHL E3 ubiquitin ligase in podocytes [272]. VHL is tumor-suppressor protein [273]. But whether this regulation exists in cancer cells is unclear. Fructose-1,6-biphosphatase (FBP1) is a rate-limiting enzyme of gluconeogenesis. MAGE-TRIM28 mediated ubiquitination and degradation of FBP1 in hepatocellular carcinoma promotes Warburg effect and cancer progression [274]. Phosphoenolpyruvate carboxykinase 1 (PEPCK1) is another rate-limiting enzyme in gluconeogenesis. High glucose in diabetes stimulates PEPCK1 acetylation, which promotes UBR5 mediated ubiquitination and degradation of PEPCK1 [275]. Studies have found that GLUT1 can be ubiquitinated for degradation in diabetes [276]. E3 ubiquitin ligase Malin mediated ubiquitination of glycogen debranching enzyme (AGL) is associated with Lafora and Cori’s disease [277]. But whether ubiquitination of GLUT1, PEPCK1 and AGL participates in tumor progression is still unknown. What’s more, ubiquitination and deubiquitination of other cancer associated metabolic enzymes is still unknown, such as enolase in glycolysis, isocitrate dehydrogenase in the TCA cycle, glycogen phosphorylase in glycogen metabolic process, and pyruvate carboxylase in gluconeogenesis. The relationship between their specific E3 ligases and DUBs and oncogenesis still need exploration.

### Ubiquitination and fatty acid metabolism

Fatty acids synthesis is necessary for membrane biosynthesis in proliferating tumor cells. CUL3-KLHL25 E3 ligase can inhibit lipid synthesis and tumor growth by targeting ATP citrate lyase (ACLY) for ubiquitination and proteolysis [278]. However, USP13 and USP30 can mediate deubiquitination of ACLY, increasing the stability of ACLY to promote development of ovarian cancer and hepatocellular carcinoma, respectively [271, 279]. TRB3-COP1 mediates proteolysis of acetyl-coenzyme A carboxylase (ACC) in ubiquitin-dependent manner, inhibiting fatty acid synthesis and stimulating lipolysis [280]. But in breast cancer cells, ACC-alpha (ACCA) interacts with AKR1B10, which prevents ACCA from ubiquitination and proteolysis, thereby promoting de novo fatty acid synthesis and enhancing tumor growth [281]. E3 ligase COP1 can ubiquitinate fatty acid synthase (FASN) with SHP2 as an adapter [282]. E3 ligase SPOP mutation, which is common in prostate cancer, inhibits its ubiquitination of FASN. Increased FASN triggers lipid accumulation and promotes prostate cancer progression [283]. AKT activation promotes deubiquitination of FASN by the USP2a and increased lipogenesis, which promotes hepatocarcinogenesis [284, 285].

3-hydroxy-3-methylglutaryl-coenzyme A reductase (HMGCR) is a rate-limiting enzyme in cholesterol biosynthesis. E3 ligases UBXD, RNF145 and RNF45 can mediate sterol-induced ubiquitination and degradation of HMGCR, attenuating cholesterol biosynthesis [286–288]. Hypoxia triggered upregulation of insulin-induced gene 2 can interact with HMGCR and promote its ubiquitination and degradation to avoid unnecessary oxygen consumption [289]. Dysregulated cholesterol metabolism is observed in multidrug resistant cancer cells (MDR), in which decreased E3 ligase RNF139 upregulates HMGCR and induces enhanced cholesterol synthesis [290]. Squalene epoxidase (SQLE), which catalyzes the first oxygenation reaction of cholesterol biosynthesis, can be ubiquitinated and degraded by E3 ligase MARCH6 under stimulation of sterol [291]. Additionally, E3 ligase MYLIP can modulate cellular cholesterol uptake by ubiquitinating LDL receptor which is responsible for cholesterol import [292]. But whether ubiquitination of SQLE and LDL receptor participate in cancer progression is unknown. In addition, ubiquitination and deubiquitination of other enzymes participating in fatty acid metabolism, such as Carnitine O-palmitoyltransferase 1 (CPT1), and their relationship with cancer progression have not been studied yet.

### Ubiquitination and amino acid metabolism

In cancer cells, glutamine serves as another important carbon source for the TCA cycle to sustain mitochondrial ATP production [1]. Glutamine uptake increases dramatically in cancer cells. NEDD4L-depleted cancer cells have enhanced neutral amino acid transporter B (ASCT2) stability and glutamine uptake to fuel the mitochondrial metabolism [293]. Promotion of RNF5-targeted ubiquitination and degradation of glutamine carrier proteins ASCT2 and SLC38A2 can improve responsiveness of breast cancer cells to Paclitaxel treatment [294]. Glutamine can be converted via GLS to glutamate, which can subsequently be converted to α-ketoglutarate to fuel the TCA cycle. Succinylation of GLS suppresses its K48-linked ubiquitination and degradation, stabilizing GLS and promoting glutaminolysis in cancer cells [295]. Study also found the function of supranutritional dose of selenite in suppressing tumor progression by promoting APC/C-CDH1 mediated GLS1 ubiquitination and degradation [296]. Ubiquitination of other enzymes involved in glutamine metabolism, such as glutamate dehydrogenase 1 (GLUD1), hasn’t been studied.

3-phosphoglycerate, an intermediate product of glycolysis, can be converted to serine by D-3-phosphoglycerate dehydrogenase (PHGDH). This conversion is subsequently associated with formate production for nucleotide synthesis. Downregulated PARKIN in cancer suppresses ubiquitination of PHGDH and enhances its stability and protein level, thereby activating serine synthesis and promoting cancer progression [297]. Serine hydroxymethyltransferase 1 (SHMT1) is involved in the conversion of serine to glycine. K48-linked ubiquitination of SHMT1 mediates its degradation in the cytoplasm. K63-linked ubiquitination of SHMT1 by UBC13 in the nucleus promotes its nuclear export and prevents it from degradation, promoting tumor progression [298]. What’s more, dysregulation of aspartate and arginine metabolism is also associated with cancer progression [299]. However, ubiquitination and deubiquitination of the enzymes participating in aspartate and arginine metabolism haven’t been studied and are worth attention in the future.

## Conclusions

In the past decades, extensive efforts have been made to clarify the molecular mechanisms associated with metabolic reprogramming in cancer. In this review, we highlight the roles of ubiquitination and deubiquitination as modulators of cancer metabolism. Facing metabolic stresses, such as hypoxia, ubiquitination and deubiquitination in cancer cells can be abnormally regulated [10, 270]. On the other hand, dysregulated ubiquitination and deubiquitination play nonnegligible roles in cancer metabolism by involving in the regulation of metabolic reprogramming related signaling pathways, transcription factors as well as metabolic enzymes. For instance, hypoxia induces E3 ubiquitin ligase SIAH2 mediated ubiquitination and proteolysis of α-KGDH, inhibiting glutamine oxidation and promoting glutamine-dependent lipid synthesis to promote tumor growth [270]. Therefore, the interactions between ubiquitination/deubiquitination and cancer metabolism are complex and require more studies. Most studies have focused on the involvement of ubiquitination and deubiquitination in the regulation of signaling pathways and transcription factors, while ubiquitination and deubiquitination of the enzymes involved in glucose, fatty acid and amino acid metabolism are worth more attention in the future.

In the regulation of cancer metabolism and tumor progression, the E3 ubiquitin ligases/DUBs-substrates network is of high complexity. Single E3 ubiquitin ligase or DUB can target numerous substrates, and one molecule can be regulated by multiple E3 ubiquitin ligases or DUBs. For example, FBXW7 acts as a tumor suppressor by targeting mTOR, HIF-1α, c-Myc and SREBP1 for degradation [43, 136, 160, 222]. However, when facing DNA damage, elevated FBXW7 mediates proteasomal degradation of p53, leading to radiotherapy resistance [300]. Amino acids can stimulate subcellular localization of TRAF6 to lysosomes for subsequent K63-linked polyubiquitination and activation of mTOR signaling [42]. However, in starvation induced autophagy, TRAF6 mediates K63-linked polyubiquitination of ULK1, which leads to stabilization of ULK1 and activation of autophagy [225]. Therefore, E3 ligases and DUBs act in a context-dependent manner. Their exact roles in cancer may vary according to their substrates, tissues types, tumor stages, or different metabolic conditions. The study of ubiquitination and deubiquitination in cancer still has a long way to go. For example, whether metabolite levels within cancer cells act as modulators of ubiquitination is ambiguous. Importantly, development of specific drugs that disrupt or enhance specific E3 ligases/DUBs-substrates interactions holds promise for more efficient and less toxic therapeutics.

What’s more, we have observed that decreased ubiquitination and increased stability of the metabolic related molecules, such as PDK1, NRF2, ULK1 and phosphoglycerate kinase 1, are associated with chemoresistance in various cancers. Thereby, modulating the activity of E3 ligases or DUBs could be exploited as a potential strategy for controlling chemoresistance in cancer treatment. Furthermore, various E3 ligases and DUBs have been already identified as potential targets for cancer therapy. Actually, many E3 ligases serve as tumor suppressors by catalyzing ubiquitination and degradation of metabolic related proteins which play oncogenic roles in cancers, indicating that drugs enhancing activities or expression of these E3 ligases should also be emphasized in further researches.

In conclusion, ubiquitination and deubiquitination are suggested to be essential regulators of metabolic reprogramming in cancer cells, demanding more studies in the future with the aim of improving cancer therapy.

## Data Availability

All the data obtained and/or analyzed during the current study were available from the corresponding authors on reasonable request.

## Abbreviations

LDH-A: L-lactate dehydrogenase A chain
mTORC1: Mechanistic target of rapamycin complex 1
HIF-1: Hypoxia-inducible factor 1
SREBP: Sterol regulatory element-binding protein
TCA: Tricarboxylic acid
AMPK: 5′-AMP-activated protein kinase
ROS: Reactive oxygen species
NRF2: Nuclear factor erythroid 2-related factor 2
HECT: Homology to E6AP C terminus
RBR: RING-between-RING
RING: Really interesting new gene
DUB: Deubiquitinating enzyme
USP: Ub-specific protease
UCH: Ub C-terminal hydrolase
OUT: Ovarian tumor protease
MJD: Machado-Josephin domain protease
JAMM: JAB1/MPN+/MOV34
DYRK2: Dual specificity tyrosine-phosphorylation-regulated kinase 2
IDH1/2: Isocitrate dehydrogenase1/2
TSC: Tuberous sclerosis complex
PDK1: 3-phosphoinositide-dependent protein kinase 1
PTEN: Phosphatase and tensin homolog
GLUT1: Glucose transporter type 1
HK1: Hexokinase 1
PDK: Pyruvate dehydrogenase kinase
AMBRA1: Activating molecule in BECN1-regulated autophagy protein 1
PFK: Phosphofructokinase
PFKL: PFK liver type
PFKP: PFK1 platelet isoform
PFKFB3: 6-phosphofructo-2-kinase/fructose-2,6-bisphosphatase 3
GLS1: Glutaminase 1
PKM2: Pyruvate kinase M2
PGK1: Phosphoglycerate kinase 1
PGAM5: Phosphoglycerate mutase 5
α-KGDH: α-ketoglutarate dehydrogenase
G6PD: Glucose-6-phosphate dehydrogenase
PEPCK1: Phosphoenolpyruvate carboxykinase 1
PDH: Pyruvate dehydrogenase
FBP1: Fructose-1,6-biphosphatase
AGL: Glycogen debranching enzyme
ACLY: ATP citrate lyase
ACC: Acetyl-coenzyme A carboxylase
FASN: Fatty acid synthase
HMGCR: 3-hydroxy-3-methylglutaryl-coenzyme A reductase
SQLE: Squalene epoxidase
CPT1: Carnitine O-palmitoyltransferase 1
MDR: Multidrug resistant
ASCT2: Neutral amino acid transporter B
PHGDH: D-3-phosphoglycerate dehydrogenase
SHMT1: Serine hydroxymethyltransferase 1
